# The Foreign Journals: Medical Statistics

**Published:** 1842-10

**Authors:** 


### MEDICAL STATISTICS.

*Statistics of Delirium Tremens in Berlin.*

FROM November 1, 1840, to November 1, 1841, 106 patients suffering from delirium tremens, were admitted into the Charité at Berlin, of whom 24, or 1 in 5 died. Of these 106, 10 had been admitted once before for the same affection, 3 twice, 2 thrice, 2 four times, 1 five times and 1 twelve times before. Most stated that they were in the custom of drinking half a pint of spirits daily, but some confessed to a quart, and one woman, only 22 years old, said that she was in the practice of drinking three pints of spirits every day. Forty-five of the patients were day labourers, 41 were mechanics, 6 were women, and 6 were persons who were above the poorer classes. It is evident, however, that most were of that class whose opportunities for indulging their vice would be greatly curtailed by the imposition of a heavy tax upon spirits.

*Allgemeine Medicin. Central Zeitung,* 9 *April,* 1842.

